# Dual Wire Technique for Transseptal Passage of Intracardiac Echocardiogram Probe During Left Atrium Appendage Closure

**DOI:** 10.1016/j.shj.2022.100020

**Published:** 2022-04-04

**Authors:** Luis Augusto Palma Dallan, Sung-Han Yoon, Steven J. Filby

**Affiliations:** Harrington Heart & Vascular Institute, University Hospitals Cleveland Medical Center, Cleveland, Ohio, USA

**Keywords:** Electrophysiology, Interventional cardiology, Left atrial appendage, Stroke, WATCHMAN

A single transseptal puncture can allow for passage of both the delivery sheath and the intracardiac echocardiogram (ICE) probe during left atrial appendage closure (LAAC). With this approach, the ICE probe is carefully directed through a dilated septal tract. However, the passage of the ICE probe through this tract can be difficult. We have previously reported a technique to facilitate transseptal passage of the ICE catheter using a snare system.^1^ We now present another solution using 2 wires to facilitate transseptal ICE passage.

We describe an 82-year-old male with multiple medical issues, including paroxysmal atrial fibrillation, heart failure with reduced ejection fraction, chronic kidney disease, history of gastrointestinal bleeding, and gait instability. His CHA_2_DS_2_-VASc score = 4 and HAS-BLED score = 4. The patient was referred for LAAC. Computed tomography angiography was used for device sizing and preprocedural planning (Figure 1). Intravenous midazolam and fentanyl were administered for conscious sedation. Access was obtained in the common femoral vein with micropuncture technique under ultrasound guidance, and 2 8F sheaths were placed. Heparin was administered intravenously, and an activated clotting time >250 seconds was documented. An 8F AcuNav ICE probe was advanced through 1 sheath, and a VersaCross transseptal sheath advanced through another. Transseptal puncture was accomplished using an NRG radiofrequency (Baylis Medical) transseptal system with ICE. Once across the septum, the VersaCross sheath was removed. The WATCHMAN double-curve access sheath was then advanced over the Baylis wire to dilate the septal tract and then withdrawn into the inferior vena cava.

**Figure 1:**
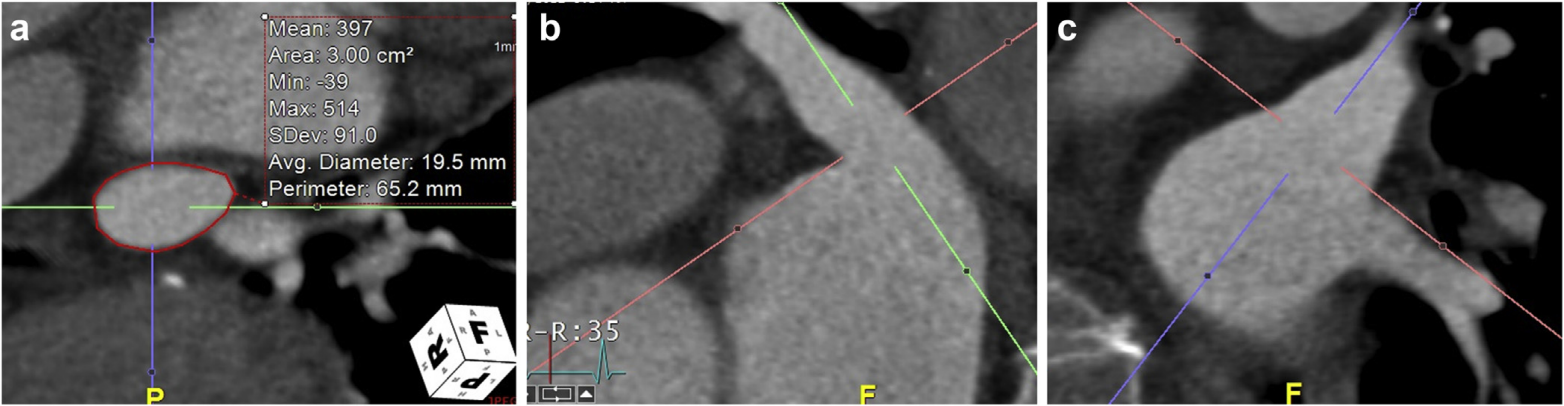
**Baseline computed tomography angiography (CTA) measurements of the left atrial appendage (LAA).** (a) Axial view; (b) Sagittal view; (c) Coronal view.

After initial attempts at passing the ICE probe through the septal tract were unsuccessful, we theorized that placing a second 0.035” wire might facilitate ICE passage by propping open the tissue. To do this, we readvanced the double-curve sheath into the left atrium and removed the dilator. We then introduced a Super Stiff J-tip Amplatz guidewire 0.035” × 180 cm alongside the Bayliss wire and withdrew the sheath back into the inferior vena cava, leaving both wires in place. The ICE catheter was then directed at the intersection of the 2 wires and able to cross through the septum (Figure 2, Panel a and b; Supplemental Video 1). Once the probe was in the left atrium, the Amplatz guidewire was removed (Figure 2, Panel c), the dilator was placed back inside the double-curve sheath, and the sheath redirected into the left atrium. The remaining case proceeded as per routine. Angiography of the left atrial appendage was performed using a 6F pigtail catheter. WATCHMAN FLX sizing was confirmed by ICE and angiography, and a 27 mm WATCHMAN FLX occluder device was placed using ICE and fluoroscopy (Figure 2, Panel d). No color Doppler flow around the device was appreciated, and 20% device compression was noted. After having satisfied P.A.S.S. (position, anchoring, size, and seal) criteria, the device was deployed. Hemostasis was achieved by the deployment of VASCADE closure devices. The patient was discharged home on the same day without incident.

**Figure 2:**
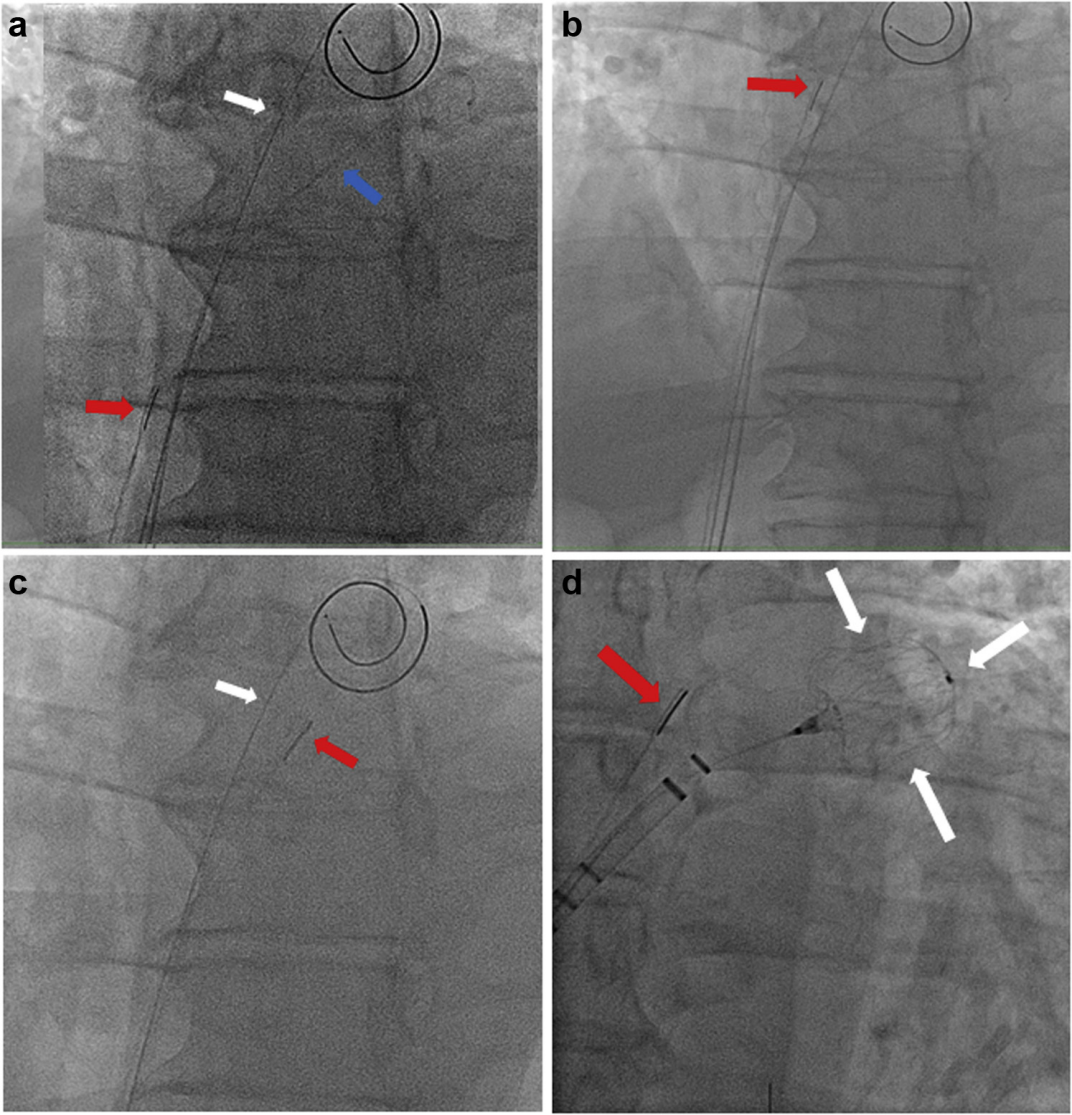
**Angiography showing the dual guidewire technique to cross the intracardiac echocardiogram (ICE) probe through the transeptal puncture for left atrium appendage closure (LAAC).** (a) ICE probe (red arrow), Bailys wire (white arrow) and Amplatz guidewire (blue arrow). (b) ICE probe (red arrow) freely inside the left atrium. (c) Bailys wire (white arrow) and ICE probe (red arrow) freely inside the left atrium after removal of the Amplatz guidewire. (d) Angiography showing successful final result and confirming the good position and compression after deployment of the WATCHMAN device. White arrows: LAA WATCHMAN closure device; Red arrow: ICE probe.

LAAC is increasingly performed for stroke risk reduction in patients with atrial fibrillation.^2^, ^3^, ^4^ Many operators choose to use a single transseptal puncture for this procedure. We have previously reported a novel technique to facilitate transseptal ICE delivery using a snare device.^1^ We now present a second technique to be used when the operator encounters such difficulty. With a wire (Super Stiff J-tip Amplatz guidewire 0.035” × 260 cm) that is readily available in most catheterization laboratories, the procedure was completed without performing a second transseptal puncture.

## Consent Statement

Consent was obtained from the patient for publication of this report and any accompanying images.

## Funding

The authors have no funding to report.

## Disclosure statement

The authors report no conflict of interest.

## Supplementary Material

Supplemental Video 1 Angiography showing the novel dual guidewire technique to cross the intracardiac echocardiogram (ICE) probe through the transeptal puncture for left atrium appendage closure (LAAC).
